# Improved Biolistic Transfection of Hair Cells

**DOI:** 10.1371/journal.pone.0046765

**Published:** 2012-10-01

**Authors:** Hongyu Zhao, Matthew R. Avenarius, Peter G. Gillespie

**Affiliations:** Oregon Hearing Research Center and Vollum Institute, Oregon Health and Science University, Portland, Oregon, United States of America; Kitasato University, Japan

## Abstract

Transient transfection of hair cells has proven challenging. Here we describe modifications to the Bio-Rad Helios Gene Gun that, along with an optimized protocol, improve transfection of bullfrog, chick, and mouse hair cells. The increased penetrating power afforded by our method allowed us to transfect mouse hair cells from the basal side, through the basilar membrane; this configuration protects hair bundles from damage during the procedure. We characterized the efficiency of transfection of mouse hair cells with fluorescently-tagged actin fusion protein using both the optimized procedure and a published procedure; while the efficiency of the two methods was similar, the morphology of transfected hair cells was improved with the new procedure. In addition, using the improved method, we were able to transfect hair cells in the bullfrog sacculus and chick cochlea for the first time. We used fluorescent-protein fusions of harmonin b (USH1C) and PMCA2 (ATP2B2; plasma-membrane Ca^2+^-ATPase isoform 2) to examine protein distribution in hair cells. While PMCA2-EGFP localization was similar to endogenous PMCA2 detected with antibodies, high levels of harmonin-EGFP were found at stereocilia tapers in bullfrog and chick, but not mouse; by contrast, harmonin-EGFP was concentrated in stereocilia tips in mouse hair cells.

## Introduction

Hair cells are specialized epithelial sensory cells in the inner ear, transducing mechanical stimuli into electrical signals in the process of hearing and balance [1]. Mechanoelectrical transduction occurs within the hair bundle, the unique organelle structure on the apical end of a hair cell. Many proteins critical for mechanotransduction, such as plasma membrane Ca^2+^-ATPase (PMCA) and harmonin, reside at discrete bundle locations that dictate their roles in mechanotransduction [2], [3]. Although many proteins have been identified in bundles by mass spectrometry [4], precise locations of many of them remains unknown. In many cases, immunocytochemistry works well to localize proteins; this method depends on antibodies of high specificity and affinity, however, which are not always available. While transient transfection of hair cells with fluorescent fusion proteins provides an alternative method that is relatively quick and independent of antibodies, most transfection methods have failed for hair cells [5].

Biolistic transfection (“gene gun transfection”) has resulted in successful delivery of foreign DNA to cells when other methods have failed [6]. In this method, gold or tungsten particles are coated with DNA, and the particulate complexes are accelerated to ultrasonic speed. The momentum of the DNA-coated particles enables them to penetrate cell membranes and enter cell nuclei, resulting in transfection of the cell [7]. This method is technically simple, requires less time for reagent generation than viral methods, and provides a quick turnaround. However, not only is the method inconsistent in its efficiency, but the shock wave and particle shower can damage the target tissue. One is usually faced with the dilemma of sacrificing penetrating power in order to avoid excessive tissue damage.

Bullfrog and chick hair cells are useful for studying protein localization in hair bundles. These cells are easy to obtain, easy to dissect, and, in the case of bullfrog, have much larger bundles that allow better imaging. Transient transfection of bullfrog hair cells using biolistic approaches has not been successful, however, due to the sacculus' large hair bundles and thick cuticular plate, the actin-rich structure immediately under the apical plasma membrane; both present obstacles to delivery and demand more penetrating power from the DNA-coated particles. Unfortunately, increasing penetrating power also greatly increases tissue damage.

Efficient transfection of mouse hair cells is also an important goal. The mouse cochlea is closely related to the human cochlea and many available mutant mouse models mimic human hearing loss well. While biolistic transfection of mouse hair cells has been successful [8], damage to hair bundles from the procedure limits the utility of this approach.

Using an improved biolistics method with more penetrating power and minimal tissue damage, we show transient transfection of hair cells from bullfrog sacculi and chick cochlea, each for the first time. Harmonin-EGFP localizes to unexpected locations in these hair cells. In addition, due to the increased penetration power and minimal blast effect of the method, we were also able to transfect mouse cochlea hair cells by shooting particles through the basilar membrane, on the opposite side of the hair cell from the hair bundle, thus minimizing the chance of bundle damage. Harmonin-EGFP expressed in these hair cell is transported to stereocilia tips, the endogenous location of harmonin. Moreover, the new method allows transfection of mouse cochlea hair cells from the back, reducing damage to hair bundles occurring during the experiment. Our biolistics method thus offers an improved method to study protein localization and function in hair cells.

## Results

### Alternative transfection methods

While trying to transfect hair cells from the bullfrog sacculus, we tried a variety of lipid- and polymer-based methods, including Lipofectamine, branched polyethyleneimine (PEI), linear PEI, Fugene, Effectene, and Superfect; in each case, we were unable to transfect hair cells (each n≥3). The lack of success may be due to physical barriers to delivery; basolateral sides of hair cells are sealed off by tight junctions made with neighboring supporting cells, preventing access to DNA complexes. Unfortunately, methods designed to open tight junctions (e.g., chelating Ca^2+^ ions with EGTA or use of solutions with reduced osmolarity) either resulted in hair cell death or no transfection. Several unique properties of the hair cells' apical surface may have also limited transfection. First, the cuticular plate forms a rigid support under the plasma membrane. Second, surrounding this cuticular plate is a ring-shaped “pericuticular necklace” [9], a zone of active exo- and endocytosis. Vesicles in this region are only about 100 nm [10], however, which is much smaller than most DNA complexes used for transfection. Because DNA complexes used in most lipid- and polymer-based transfection methods are larger than 100 nm [11], [12], they probably cannot enter hair cells by apical endocytosis. The efficiency of in vitro transfection increases with the size of DNA complex [11], [13], [14]; such DNA complexes usually enter cells through phagocytosis, which can accommodate much larger particles [15]. Although DNA complexed to polyethyleneimine (PEI) can apparently be made into particles smaller than 100 nm [11], [16], [17], we were unable to transfect bullfrog hair cells or COS-7 cells with PEI-DNA complexes (n = 3). To specifically trigger PEI-DNA endocytosis, we also chemically crosslinked the transferrin receptor (TfR) to PEI, then formed a TfR-PEI-DNA complex [18]. Unfortunately, that strategy also did not yield any transfected bullfrog hair cells (n = 3).

In vitro and in vivo electroporation methods have been successfully used to transfect developing mouse and rat hair cells or hair-cell progenitors [19]–[21]. We also tried in vitro electroporation with bullfrog sacculi, which again resulted in no transfection of hair cells or indeed any cells in the epithelium (n = 5). The lack of success likely arose because of the specialized apical surface of the adult hair cell; high voltages led to total destruction of the hair cell, while lower voltages did not permit entry of DNA (n = 3). Finally, when we combined electroporation with loosening tight junctions with low Ca^2+^ and incorporation of DNA into a complex with PEI, no hair cells were transfected (n = 3).

### Biolistic transfection

Using standard protocols and unmodified instruments, we attempted transfection of hair cells in the bullfrog sacculus with two biolistic instruments from Bio-Rad, the PDS-1000 and the Helios Gene Gun. We were unable to transfect bullfrog hair cells using either system, while both systems permitted low-efficiency hair-cell transfection in chick utricles, mouse utricles, and mouse cochlea [8], [22]. When we used conditions that resulted in higher particle speed, we occasionally saw a few cells transfected in the non-sensory region of the sacculus. Under those conditions, however, the increased shockwave generated from either system destroyed nearly all of the hair bundles. We reasoned that as we increased helium pressure, which increases gold-particle velocity, the increased shockwave destroyed the hair cells, especially their bundles, which face the direction of gold particles.

Multiple groups have tried to modify the PDS-1000 or the Helios Gene Gun to achieve better transfection efficiency. For example, Thomas and collaborators described a modification to transfect fragile insect tissues [23], while O'Brien's group described a modification of the Helios Gene Gun that improved penetrating power several-fold [24]. After modifying our PDS-1000 according to Thomas et al., we were still unable to transfect bullfrog hair cells, although we occasionally saw cells transfected in the non-sensory region. Poor transfection probably resulted from insufficient penetrating power, as the method was aimed at minimizing tissue damage. We also obtained the modified Helios Gene Gun part described by O'Brien and although this component allowed generation more penetrating power by directing the helium stream with a focusing nozzle, it also substantially increased shockwave damage to the tissue.

**Figure 1:**
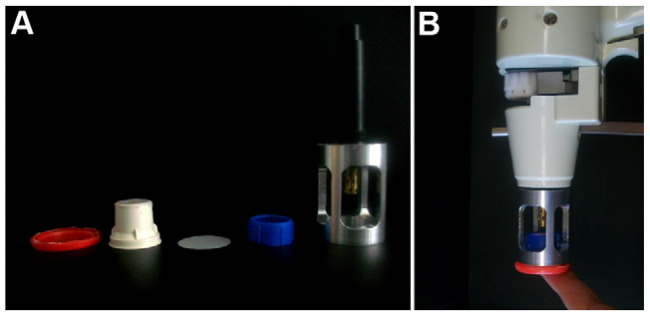
Diagram of modifications to the Helios Gene Gun. *A*, components of the modified setup. From left to right, SARSTEDT 50 ml conical tube cap, with sides cut; 15 mm Netwell Insert (with 74 µm mesh size polyester membrane); diffuser screen; plastic ring holder to fix the diffuser screen on top of the Netwell insert; metal adaptor that connects the Gene Gun to the sample chamber. *B*, assembled setup.

We therefore focused on reducing the shockwave from the O'Brien method while preserving its penetrating power. To reduce the shockwave, we shortened the focusing nozzle and put the sample in a semi-air-tight chamber, with porous polyester mesh and diffusion screen with small holes on top. The sample chamber is made of: (1) a SARSTEDT 50 ml conical tube cap at the bottom, where sample to be transfected is placed; (2) a 15 mm Netwell Insert (with 74 µm mesh size polyester membrane), inverted and trimmed to fit into the tube cap; (3) a diffuser screen (5 µm pore size) placed on top of the inverted Netwell insert; and (4) a plastic ring holder to fix the diffuser screen on top of the Netwell insert. Metal adaptors that connect the Gene Gun to the sample chamber were used to precisely control the sample position relative to the tip of the nozzle and distance between sample and tip of the nozzle; we used three different adaptor lengths, corresponding to three different tip-to-sample distances (Fig. 1).

**Figure 2:**
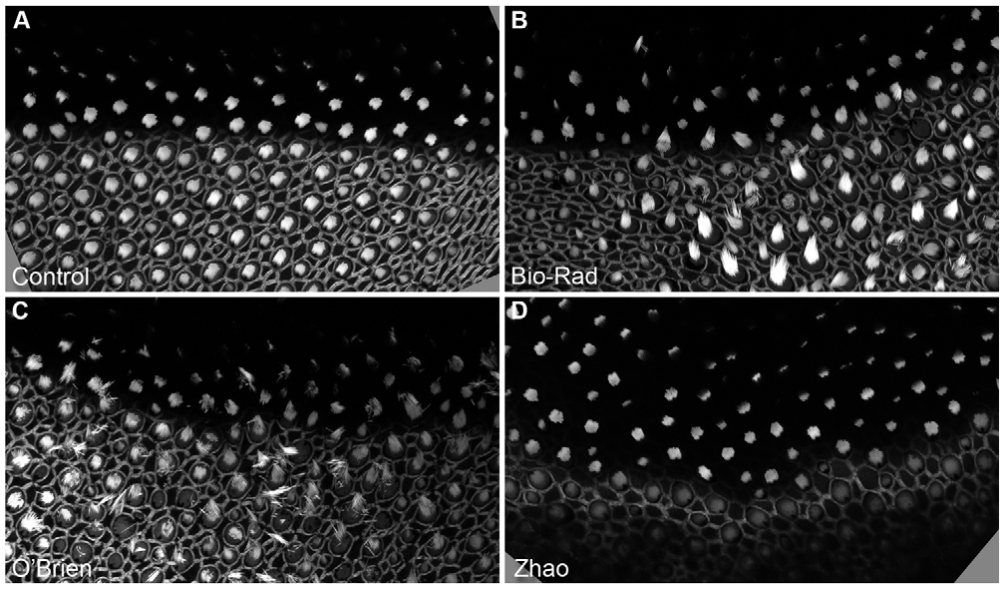
Shockwave damage from biolistics transfection methods. Bullfrog sacculi were dissected, shot with different Gene Gun setups, immediately fixed, and stained with Alexa Fluor 488-phalloidin. *A*, control (not shot); B, standard Bio-Rad setup, at 200 psi; C, O'Brien setup, 75 psi; D, Zhao setup, 200 psi. Panels are 950 µm wide.

**Figure 3:**
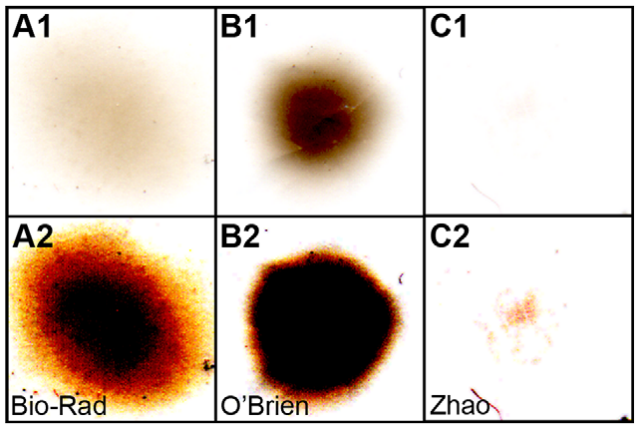
Particle pattern of different biolistics transfection methods. Millipore diffuser membranes (3 µm) were placed at the sample target position for shooting. After shooting, the diffuser membranes were scanned using a regular flatbed scanner. *A1*–*C1*, particle patterns of standard Bio-Rad setup, O'Brien setup, and Zhao setup, respectively. *A2*–*C2*, images with increased digital gain showing the much-reduced particle density in Zhao setup (C2). Panels are 6.9 mm wide.

The shortened nozzle tip and the semi-air-tight chamber, with a net-supported membrane on top, greatly reduced the shockwave; sacculi were not disturbed even at 200 psi, while the standard Bio-Rad and O'Brien set-ups blew the sacculi away or damaged the hair cells badly. Excessive particles also cause tissue damage [25]. Because we used two layers of filters, one with a 74 µm mesh and one with 5 µm pores, we substantially reduced the particle density as compared to the standard Bio-Rad and O'Brien methods. We estimated hair-cell damage using the three methods by labeling saccular actin with phalloidin (Fig. 2). Damage to hair bundles was evident with both the Bio-Rad and O'Brien methods; bundles were missing, knocked over, or splayed (Fig. 2B–C). By contrast, using the present method, bundles remained vertical and appeared intact (Fig. 2D). With this method, particle density was dramatically reduced (Fig. 3), minimizing the damage caused by particles themselves. Because of the much reduced particle density, we were unable to directly compare penetration power of the three methods; it is likely, however, that some particles pass through the two filters at high velocity.

Using this modified setup, we were able to transfect hair cells of the mouse cochlea, bullfrog sacculus, chick utricle, and chick cochlea with proteins that localize to hair bundles.

### Transbasilar-membrane transfection of mouse hair cells

**Figure 4:**
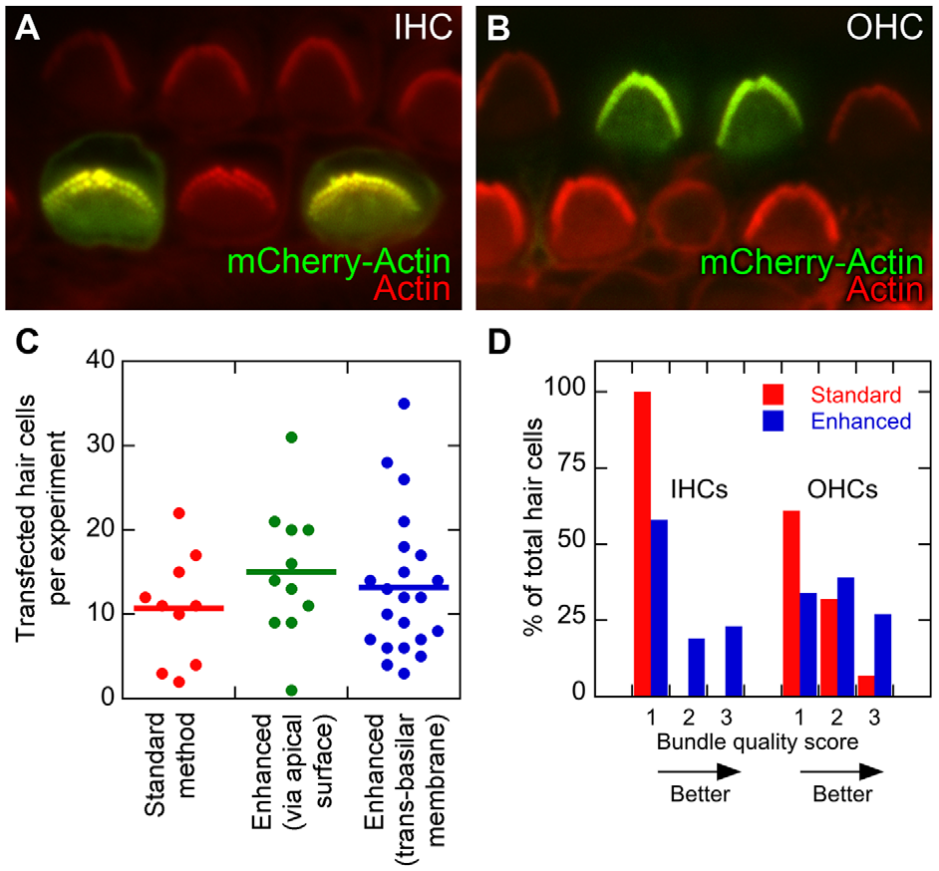
Transfection efficiency for mCherry-actin in mouse cochlear hair cells. *A*, Two inner hair cells (IHC) transfected with mCherry-actin. mCherry-actin is pseudocolored green, while phalloidin-stained actin is shown in red. *B*, Two outer hair cells transfected with mCherry-actin. Panel width in A and B is 30 µm. *C*, Transfection efficiency for standard method and enhanced method. The enhanced method was used for either apical or basal delivery. Horizontal lines indicate mean number of transfected cells per experiment. *D*, Morphological quality of hair bundles using the standard and enhanced (basal delivery) methods. Bundles from transfected hair cells were scored with a three-point scale, with 1 corresponding to a substantially disrupted bundle and 3 corresponding to a bundle that did not appear disruption.

Because of increased penetrating power, we suspected that DNA-coated gold particles might be capable of accessing cochlear hair cells from the back side of the basilar membrane. With a coarser diffuser membrane (12 µm pore size instead of 5 µm), we were able to transfect mouse cochlea hair cells through the basilar membrane. Since the gold particles enter the hair cells from the basal side, damage to the hair bundle by the gold particles and blast was minimized. Removal of the tectorial membrane for transfection is unnecessary, leaving hair bundles protected from mechanical damage, including liquid surface tension during transfer of the organs. Transbasilar-membrane gene gun delivery of mCherry-actin plasmids successfully led to transfection of mouse cochlear hair cells (Fig. 4A, B).

We compared three methods for transfection of mouse hair cells: (1) the original method, as described by Belyantseva [8]; (2) the present method, using a 5 µm diffuser membrane, from the apical surface; and (3) the transbasilar-membrane method. All three methods gave roughly similar transfection efficiencies of 10–15 hair cells per cochlea (Fig. 4C) [21].

To assess the consequences of transbasilar-membrane transfection on hair-bundle structure, we qualitatively scored transfected hair cells with respect to the morphological status of their bundles from a score of 1, with an absent or severely damaged bundle, to a score of 3, where the bundle is intact. The proportion of inner hair cells that exhibited intact bundles using the transbasilar-membrane method was roughly 25% while the remaining bundles showed clear defects (Fig. 4D). By contrast, with the standard method, all of the bundles from inner hair cells were damaged to some degree. Outer hair cells were also transfected with minimal damage. Using the enhanced method, nearly 20% of transfected outer hair cells retained intact hair bundles, similar to the proportion seen in inner hair cells (Fig. 4D). Only a few outer hair cells transfected with the standard method had intact bunldes. While the fraction of transfected hair cells yielding intact bundles remains relatively low, the transbasilar-membrane method neverthless provides a substantial improvement over the previously published method (Fig. 4D).

### Transfection with other fluorescent protein fusions

**Figure 5:**
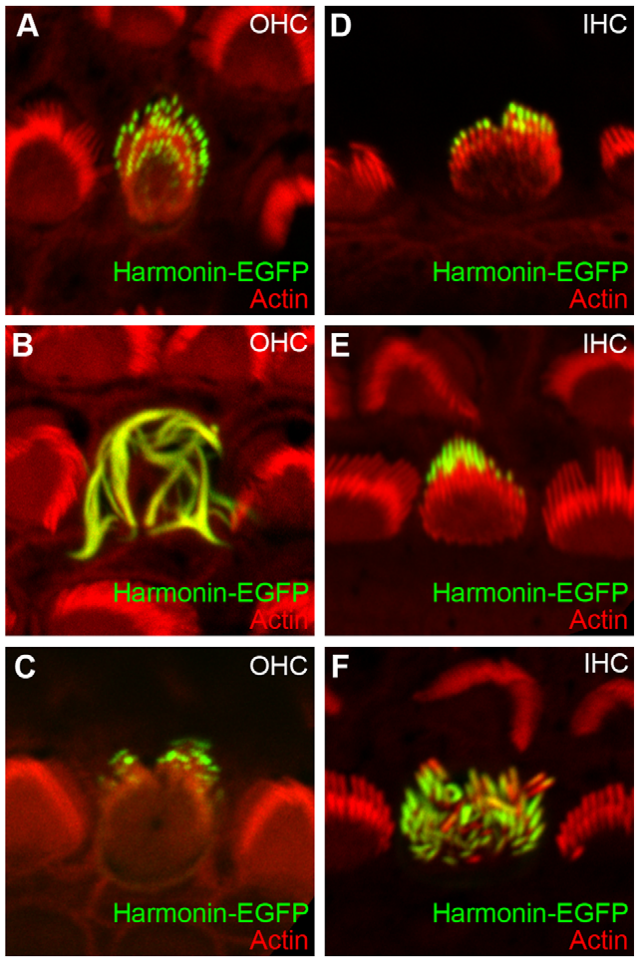
Localization of harmonin-EGFP in transfected mouse cochlea hair cells. Harmonin-EGFP (green) was transfected into cochlear hair cells of CD1 mice at P4 using the modified biolistics setup. Phalloidin-stained actin is shown in red. Harmonin-EGFP was localized close to the tips of the stereocilia of both outer (*A*–*C*) and inner (*D*–*F*) hair cells. In some cases, we saw fused stereocilia in outer hair cells, similar to harmonin-EGFP transfected hair cells in chick cochlea. Panels are 17.6 µm wide.

Having established the relative gentleness of transfection from the cochlear hair cells' basal side, we examined expression in inner and outer hair cells of a construct encoding a fusion of mouse harmonin b (official protein symbol: USH1C) and EGFP. Harmonin-EGFP was located in the upper part of the stereocilia, usually only near the tips (Fig. 5A, C–E).

**Figure 6:**
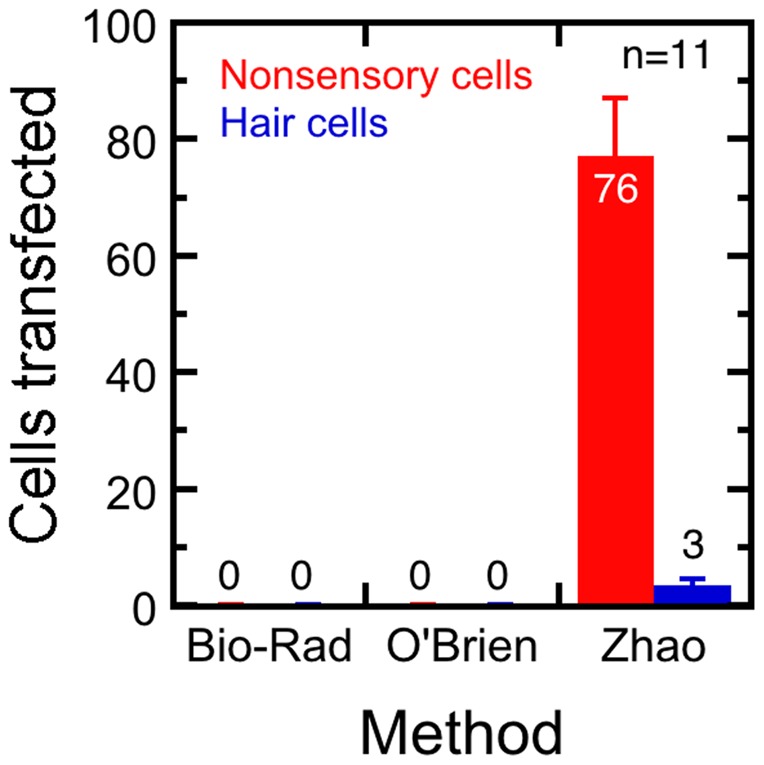
Efficiency of biolistics transfection methods in bullfrog sacculi. Bullfrog sacculi were transfected using the standard Bio-Rad setup, O'Brien setup, and Zhao setup. Sacculi were cultured, fixed and stained, then imaged. Harmonin-EGFP positive cells were counted. The average number of transfected nonsensory cells were shown in red, while the number of transfected hair cells were shown in blue. Error bars represent SEM. Approximate numbers of transfected cells per epithelium are indicated by bars, as is the total number of epithelia analyzed.

**Figure 7:**
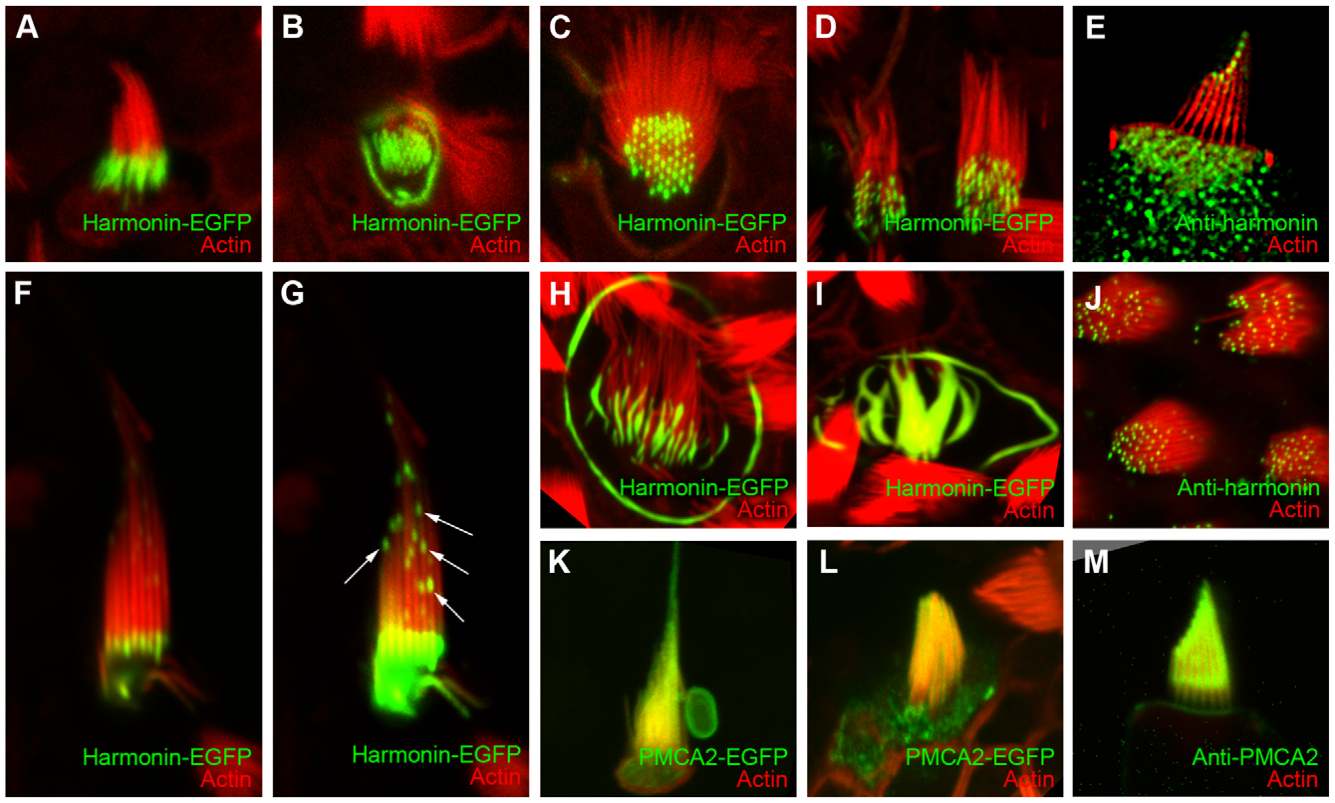
Localization of harmonin-EGFP and PMCA2-EGFP in transfected bullfrog and chick hair cells. Mouse harmonin b fused with EGFP on the C-terminus (harmonin-EGFP) and frog PMCA2 fused with EGFP on the C-terminus (PMCA2-EGFP) were transfected into frog and chick hair cells using the modified setup. Harmonin-EGFP and PMCA2-EGFP are pseudo-colored green, and phalloidin-stained actin is shown in red, except in panel K, in which co-transfected mCherry-actin is shown in red. *A*–*D*, Frog sacculus hair cells transfected with harmonin-EGFP. *E*, Frog sacculus hair cells labeled with anti-harmonin antibody H3. Structured-illumination image. *F*, Chick utricle hair cell transfected with harmonin-EGFP; *G*, Higher digital gain image of E, showing clusters of harmonin-EGFP along the stereocilia (arrows). *H*–*I*, chick cochlea hair cells transfected with harmonin-EGFP. *J*, Frog sacculus hair cells labeled with anti-harmonin antibody H3. *K*, Live-cell imaging of a chick utricle hair cell transfected with PMCA2-EGFP and mCherry-actin (red). *L*, Frog sacculus hair cell transfected with PMCA2-EGFP. M, Frog sacculus hair cell labeled with anti-PMCA2a antibody F2a. Panels are 18.4 µm wide.

With the modified setup, we were also able to transfect hair cells of frog sacculus, chick utricle, and chick cochlea with harmonin-EGFP (Figs. 6 and 7). To estimate transfection efficiency, we transfected bullfrog sacculus with EGFP fused to harmonin b (harmonin-EGFP) and observed on average three transfected hair cells in each sacculus, and many more cells outside the sensory epithelium (Fig. 6). Transfection with a plasmid encoding mCherry-actin was generally ineffective, presumably because mature hair cells like those in the adult bullfrog sacculus exhibit very little actin turnover [26].

In all three types of hair cells, harmonin-EGFP was concentrated at the hair bundle's base, in the taper region of the stereocilia. We also observed harmonin-EGFP in ring-like structure that surrounded the cuticular plate (Fig. 7B, G, H). When the image gain was increased, small, discrete clusters of harmonin-EGFP were sometimes seen along stereocilia shafts (Fig. 7F); this result suggests that harmonin is transported to the upper part of the stereocilia in a cluster and is consistent with the observation that harmonin interacts with the molecular motor myosin-VIIA harmonin and is transported towards stereocilia tips [27], [28]. In chick cochlea, we saw several transfected hair cells with what appeared to be fused stereocilia (Fig. 7H); this result is consistent with the previous finding that harmonin b can bundle actin [29]. Localization of EGFP-harmonin differed significantly from that of endogenous harmonin detected by a specific antibody, however (Fig. 7E, J). In addition, our harmonin b cDNA construct was from mouse, which could account for why harmonin-EGFP was poorly transported towards stereocilia tips in bullfrog hair cells.

We also transfected frog and chick hair cells with frog PMCA2, the plasma membrane Ca^2+^-ATPase (official protein symbol: ATP2B2) fused to EGFP. PMCA2-EGFP was concentrated in the hair bundle and was uniformly distributed along the stereocilia (Fig. 7K, L). We also observed PMCA2-EGFP in the cell body and apical surface of the hair cell. These observations are consistent with previous immunocytochemical and transfection reports [30], [31]. We also transfected frog and chick hair cells with rat PMCA2-EGFP and saw similar localization. Immunoreactivity of PMCA2 in frog and chick hair cells is characterized by reduced staining at the stereocilia taper region compared to the stereocilia shaft [2], seen in Fig. 7M. Although we did not clearly observe this pattern in transfected hair cells, we analyzed only a few cells; none of their hair bundles were visible in profile, optimal for viewing this staining pattern [2].

## Discussion

Transient transfection is useful for studying protein localization in cells. Unfortunately, hair cells have proven difficult to transfect both because hair cells are post-mitotic, limiting nuclear entry of exogenous DNA, and because entry of DNA into hair cells is strictly limited. Hair cells are surrounded by supporting cells on their basolateral sides, with tight junctions sealing the borders, such that only the apical surface is accessible in a dissected explant. Furthermore, while the apical surface is a site of active endocytosis, only very small vesicles, about 100 nm in size, are involved; this size is too small for typical DNA complexes. Moreover, the plasma membrane of the apical surface is supported by a dense actin network and is thus much more rigid.

Hair cells have been successfully transfected using in utero electroporation [20], electroporation of organotypic cultures [19], [21], adenovirus [32], lentivirus [33], adeno-associated virus (AAV) [33], and biolistics [34]. In utero electroporation in mice at E12.5 is very efficient, as more than 50% of hair cells can be transfected with GFP [20]; however, this is a very challenging technique and is not practical for most labs. Although virus-based techniques also work, all viruses have some hair-cell toxicity [35]–[38], making them less suitable for studying protein localization patterns. AAV causes the least toxicity of all tested viruses but has a small packing limit (4.5 kb total) and thus can only direct expression of small proteins. Because DNA solutions can only access the apical surface, where hair bundles reside, successful electroporation of hair cells with little damage to the hair bundle in organotypic cultures has only been reported in young mouse cochlea (earlier than P5, before hair bundles mature) and has low efficiency [21].

Although biolistic transfection with the Gene Gun is effective for transiently transfecting hair cells of some preparations, including mouse cochlea [8], hair cells in other preparations, especially bullfrog sacculus and chick cochlea, haven proven recalcitrant. We determined that a high particle speed with low shockwave is necessary to transfect them. By including a focusing nozzle, the modification of Helios Gene Gun described by O'Brien and colleagues [24], we saw a dramatic increase in penetrating power but also more tissue damage, presumably from the stronger shockwave that the focusing nozzle generated. Reducing the shockwave from the Helios Gene Gun while preserving the particle speed and hence penetrating power is challenging; it is difficult to separate the particle speed from its driving force, the shockwave. To reduce the shockwave, we shortened the nozzle and put the sample in a semi-airtight chamber, with a coarse mesh and a fine diffuser screen on top. The filters stop >90% of the particles, yet because the focusing nozzle concentrates the particles, enough particles reach the sample.

The improved penetrating power afforded by the enhanced method allowed us to transfect mouse hair cells from the basilar membrane side; because gold particles do not pass through the apical surface, where hair bundles are located, damage to bundles is minimized. Bundle damage is also decreased because hair cells' apical surfaces remain relatively protected; when mouse cochlea explants dissected with the standard protocol, which exposes the bundles, are taken through the transfection procedure without biolistic bombardment, many bundles are still damaged (M.R.A. and P.G.G., unpublished data). Thus the enhanced method with basal transfection should prove more suitable for studying transfected hair cells with minimal bundle damage.

We used the enhanced method to examine transfection of harmonin in hair cells. In mouse bundles harmonin-EGFP was located near stereocilia tips and was absent from tapers; this localization matched what is seen in acutely dissected mouse cochlea using anti-harmonin antibodies [3].

In a few hair cells from mouse and many in bullfrog and chick, high levels of harmonin-EGFP expression led to distorted harmonin- and actin-containing structures that appeared to consist of fused stereocilia (Fig. 5B, 6I). Large apical rings were also seen in bullfrog and chick (Fig. 6B, H, I). Both of these structures were reminiscent of the wavy actin bundles seen in tissue-culture cells [29]. At high levels, harmonin b bundles actin [29], which likely occurs because harmonin b contains an actin-binding site [29] and can oligomerize [39]. The distorted stereocilia structures likely occurred because of relatively high levels of harmonin expression. No structures similar to these were seen in any hair cells transfected with mCherry-actin. As in any experiment where exogenous proteins are delivered to the cell, the level of expression relative to the native protein is crucial. While the distorted structures are undoubtedly artifactual, they also point to harmonin's ability to oligomerize, which thought to be essential for function.

In bullfrog and chick hair cells, we usually saw high levels of expression at stereocilia tapers (Fig. 6A–G). When expressed in tissue-culture cells, harmonin initially localized to focal adhesions, sites of clustered actin filament barbed ends [29]. High expression at stereocilia tapers was thus surprising, since most stereocilia actin filaments' pointed ends are found at the tapers. The extent of harmonin clustering at tapers varied from cell to cell, probably reflecting differences in expression levels.

In many cells we also detected much smaller clusters of harmonin in stereocilia shafts; these clusters were usually asymmetric, with the longitudinal axis (relative to the stereocilia shaft) being 1.5– to 2-fold longer than the horizontal axis. We presume that these are small clusters in transit towards stereocilia tips; their size may reflect geometric considerations of the transport mechanism or stereocilia structure. Regardless, endogenous harmonin was only seen clearly at stereocilia tips in bullfrog hair cells, located at the tip link's upper end [3].

The difference between the species could reflect inefficient transport of the mouse harmonin fusion protein in bullfrog and chick bundles, or it could indicate differences in structure of the taper region. Adult bullfrog and E20–E21 chick hair cells are more mature than P4 mouse hair cells; the difference in harmonin-EGFP localization may also represent developmental differences in bundle structure.

In conclusion, we modified the Helios Gene Gun and developed a protocol to transfect hair cells from bullfrog sacculus and chick cochlea, previously recalcitrant to transfection. Moreover, by using this method to transfect mouse hair cells from the basilar-membrane side, we reduced damage to hair bundles from the procedure. With simple optimization steps, the protocol can be easily adapted to transfect hair cells from other species and organs and should result in increased transfection efficiency with minimal damage to hair cells. Moreover, the combination of increased penetration power and reduced shockwave will be of general applicability for transfection of many other cell types and tissues for which standard methods for foreign DNA introduction are unsuccessful. The improved gene-gun transfection method should facilitate studies examining protein localization, interaction, and function in hair cells and many other cell types.

## Materials and Methods

### Ethics statement

Animal experiments reported here were approved by the Oregon Health & Science University Institutional Animal Care and Use Committee (IACUC); the approval number was A684. All experiments began with euthanasia of the animal, carried out using methods approved by American Veterinary Medical Association Panel on Euthanasia.

### Materials

The Helios Gene Gun (1652411), PDS-1000, gold particles (1652264), Polyvinylpyrrolidone (PVP) and Tefzel tubing (1652441) were purchased from Bio-Rad; Branson Ultrasonic Cleaner (water bath) model 1210; TreffLab microcentrifuge tubes were from Scidynamics LLC; the 15 mm Netwell Insert with 74 µm mesh size polyester membrane was from Corning; the diffuser screens were from Millipore (5 µm pore size, TMTP 025000) and SPI supplies (12 µm pore size, E12025-MB); the plastic ring holder was made from the cap of Fisher culture test tube (#14-956-1J); and the 50 ml conical tube cap was from SARSTEDT; Spermidine (S4139) and anhydrous ethanol (459836) were from Sigma; Cell-tak was ordered from BD Biosciences (354240). The modified Gene Gun barrel was purchased from Dr. John O'Brien, and the tip of the focusing barrel were shortened by 5 mm in a local machine shop.

### Preparation of tubing loaded with DNA-coated gold particles (frog and chicken)

For frog and chicken transfection, 2 mg of 1.6 μm diameter gold particles (Bio-Rad) were transferred to a 1.5 ml microfuge tube; 25 μl spermidine (0.05 M in dH_2_O, pH 10.5) was added. The spermidine-gold mixture was sonicated in a bath sonicator for 20 seconds. Mix 4 µg DNA, dH_2_O, and 5 µl 25% glycerol in 12.5 µl total volume in a microcentrifuge tube. The DNA solution was then mixed with the spermidine-gold mixture and vortexed. While still vortexing, 25 μl of 1 M CaCl_2_ is added dropwise. This suspension is sonicated briefly and incubated at room temperature for 10 min. The suspension was then washed three times with 0.5 ml 100% ethanol, with brief sonication in between. The DNA coated gold particles were then resuspended in 0.6 ml of 100% ethanol.

For mouse transfection, 4.2 mg of 1.6 μm diameter gold particles were transferred to a 1.5 ml microfuge tube; 25 μl 0.05 M spermidine (diluted in 100% ethanol) was added to the gold beads, vortexed for 15 seconds and sonicated in a bath sonicator for 30 seconds. 8.3 μg of plasmid DNA constituted in a total volume of 25 μl was added to the sonicated beads. 25 μl of 1 M CaCl_2_ was added dropwise to the gold bead and DNA suspension and incubated at room temperature for 10 minutes. The suspension was then resuspended and washed three times with 0.6 ml 100% ethanol. After the final wash the supernatent was removed entirely and replaced with 0.6 ml 50 µg/ml PVP.

### Bullet preparation

Nitrogen gas (0.3–0.4 LPM) was passed through a 6-inch piece of Tefzel tubing for a total of 10 minutes. The tubing was then attached to a 2 ml syringe. After a brief sonication, the suspended DNA-gold particles were drawn into the tubing. The gold particles were allowed to settle for 10 min, before the supernatant was slowly removed using the syringe. The tubing was rotated for 30–40 sec, dried with a flow of nitrogen, cut, and stored desiccated at 4°C until use.

#### Mouse

Nitrogen gas (0.3–0.4 LPM) was passed through a 6-inch piece of Tefzel tubing for a total of 10 minutes. The tubing was then attached to a 2 ml syringe. After a brief sonication, the suspended DNA-gold particles were drawn into the tubing. The gold particles were allowed to settle for 3 min, before the supernatant was slowly removed using the syringe. The tubing was rotated for 30–40 sec, dried by applying nitrogen gas for 5 minutes, cut, and stored desiccated at 4°C until use.

### Dissection, biolistics, and organotypic cultures

Sacculi from adult bullfrogs (mixed sexes, 2–5 inch bodies) were dissected; the otolithic membrane was loosened with a 20 min treatment with 65 µg/ml protease XXIV (Sigma) at room temperature, then was then carefully removed with forceps. After shooting at 200 psi, the sacculi were washed 2x with culture medium 0.75x DMEM/F12 with HEPES (Invitrogen #11039), supplemented with 10% FBS and 20 µg/ml carbenicillin. Sacculi were then cultured for 16–24 hours at 27°C with 5% CO_2_.

Utricles and cochleas were dissected from E20–E21 chicks; otolithic or tectorial membranes were removed before shooting at 200 psi. Organs were then cultured for 16–24 hours at 37°C with 5% CO_2_ in DMEM/F12 with HEPES containing 2% FBS, 5 µg/ml carbenicillin, 0.002% ciprofloxacin [21].

Cochleae were dissected from CD1 or C57BL/6J (P4.5) mice in ice-cold PBS supplemented with 100 µM CaCl_2_ and 1 mM MgCl_2_. After exposing the cochlea by removal of the temporal bone, the organ of Corti, together with the lateral wall and Reissner's membrane, was removed from the inner ear. To carry out this step, we grasped the base of the lateral wall and organ of Corti with forceps and unwound them together as one piece from the modiolus. The tissue was oriented basilar membrane side up, all liquid was removed and the particles were delivered at 200 psi. Instead of using the standard 5 µm pore size diffuser, we use a 12 µm SPI-Pore polycarbonate membrane filter. After biolystic delivery of the particles the lateral wall was removed and the organ of Corti was attached to the bottom of a Cell-Tak coated 35 mm dished and cultured in the media containing DMEM/F12 containing 5 µg/ml carbenicillin for 24–48 hours at 37°C with 5% CO_2_.

### Confocal microscopy

After culturing, the organs were fixed with 4% formaldehyde for 30 min at room temperature, the tectorial membrane was removed and the tissue was counter-stained for 1 hr with TRITC-phalloidin or Alexa Fluor 488-phalloidin (in PBS containing 0.2% saponin), washed, and mounted. In some cases, cells were stained for harmonin using an antibody (H3) directed against the C-terminal PDZ domain [3] or with an antibody (F2a) directed against the “a” splice form of PMCA2 [30]. All samples except that of Fig. 5E were observed with an Olympus FV1000 confocal microscope equipped with a 60x, 1.42 NA oil-immersion plan apochromat objective. The image of Fig. 5E was obtained using structured-illumination microscopy with an Applied Precision OMX system.
